# *In vitro* antibacterial, anti-biofilm, anti-quorum-sensing, and cytotoxic activities of leaf crude extracts of *Cannabis* “Gorilla glue 1”

**DOI:** 10.3389/fvets.2026.1750799

**Published:** 2026-03-02

**Authors:** Shadrack Monyela, Prudence N. Kayoka, Olasunkanmi S. Olawuwo, Wonder Ngezimana, Lufuno E. Nemadodzi

**Affiliations:** 1 Department of Agriculture and Animal Health, University of South Africa, Science Campus, Johannesburg, South Africa; 2 Department of Horticulture, Faculty of Plant and Animal Sciences and Technology, Marondera University of Agricultural Sciences and Technology, MUAST Innovation & Agro-Industrial Park Campus, Marondera, Zimbabwe

**Keywords:** antibacterial, biofilm, *Cannabis*, cytotoxicity, fish bacteria

## Abstract

The resilience of biofilms makes it challenging to treat bacterial infections using conventional antibiotics. The study aimed to assess the antibacterial, anti-biofilm, anti-quorum-sensing, and cytotoxic activities of acetone extracts of *Cannabis* “Gorilla Glue 1” against fish pathogens. Antibacterial activity was determined using the two-fold serial microdilution method, while anti-biofilm activity was assessed using a modified crystal violet staining *in vitro* assay. Anti-quorum-sensing activity was evaluated via inhibition of violacein production in *Chromobacterium violaceum* (ATCC 12472). Cytotoxicity was assessed using a colorimetric assay against Vero kidney cells. Solvent extracts from treatment 0.36 g N; 0.12 g P; 0 g K showed the lowest minimum inhibitory concentration (MIC) value (0.02 mg/mL) against *Edwardsiella tarda* (ATCC 15947) and *Pseudomonas fluorescens* (ATCC 13525) compared with other treatments. All tested solvent extracts demonstrated the ability to prevent or disrupt biofilm formation; however, treatment 0.36 g N; 0.06 g P; 0.12 g K showed consistent anti-biofilm activity (>50% inhibition) against all tested pathogens. All solvent extract treatments exhibited comparable anti-quorum-sensing activity, while treatment 0.36 g N; 0.06 g P; 0.12 g K demonstrated the highest inhibition of violacein production (98.61% at 1.25 mg/mL). Most solvent extracts were non-cytotoxic to Vero cells, with LC_50_ values >0.1 mg/mL, except treatment 0 g N; 0.24 g P; 0 g K, which showed high cytotoxicity (LC_50_ = 0.04 mg/mL). Treatments 0.36 g N; 0.12 g P; 0 g K, 0 g N; 0.36 g P; 0.6 g K, and 0 g N; 0 g P; 0 g K exhibited moderate toxicity (LC_50_ = 0.06 mg/mL). Treatment 0.36 g N; 0.12 g P; 0 g K displayed the highest selectivity index (3.00) against Vero cells, indicating the most favorable safety profile among the extracts investigated. Leaf extracts of *Cannabis* exhibited useful bioactivities coupled with low cytotoxicity, providing impetus for further studies on their potential development as protective feed additives against microbial infections in fish production.

## Introduction

The farming of aquatic animals, particularly fish farming, remains the largest and fastest-growing sector in global aquaculture food production (1). A study revealed that global fisheries production reached 96 million tons in 2023, representing a 16.93% increase from previous years (2). Fish farming significantly benefits nutrition and food security by providing a rich, affordable source of protein and micronutrients, particularly in food-insecure areas, while simultaneously creating jobs and income opportunities for millions of people worldwide (3). However, this sector’s long-term viability is threatened by frequent outbreaks of bacterial diseases that affect fish and promote resistance to currently used antibiotics. Beyond the rising interest in organic seafood, the presence of zoonotic pathogens in commercial fish poses a significant public health risk to consumers, with fish often becoming contaminated through exposure to infected waters, contaminated sediments, or direct contact with pathogens in their environment (4). Thus, bacteria found in fish reflect the safety and general condition of aquatic environments. The most prevalent opportunistic bacteria involved in fish diseases belong to families of Gram-negative bacteria (GNB) responsible for severe infections (5). Notably, pathogenic bacteria such as *Aeromonas hydrophila*, *Edwardsiella tarda*, and *Pseudomonas fluorescens* are major pathogens responsible for significant disease outbreaks in fish farming (6, 7). These pathogens cause substantial yield losses, leading to major global economic setbacks (8). Efforts to treat and prevent infections caused by these pathogens have largely relied on antibiotics, and their overuse has consistently resulted in bacterial resistance (9). More than 80% of fish microbial infections are associated with bacterial biofilms (10). Biofilms are microbial communities encased in a self-produced extracellular polymeric substance (EPS) matrix, which promotes irreversible attachment to biotic and abiotic surfaces, enhances protection, and contributes to antimicrobial resistance (11, 12). These communities play a critical role in preventing antibiotic penetration and resisting harsh environmental conditions (13). Biofilms also enhance bacterial growth, antibiotic resistance, immune evasion, and genetic material transfer (14). Biofilm formation is closely linked to intercellular communication, known as quorum sensing (QS) (15). Through signaling molecules called autoinducers, gene expression is regulated, making bacterial communities difficult to eradicate using antimicrobial agents or host immune defenses (16). Consequently, there is a growing need for alternative or complementary strategies to combat antibiotic resistance associated with biofilm formation. Antimicrobial agents that inhibit pathogen growth while exhibiting minimal host toxicity are considered promising candidates for new drug development (17). Medicinal plants such as *Cannabis* have been used for centuries to treat infectious diseases (18) and are rich in bioactive compounds, including cannabinoids, tannins, alkaloids, flavonoids, and saponins, which have demonstrated antimicrobial properties *in vitro* (19). Historically and traditionally, various parts of this plant, particularly the aerial parts, have been used to treat a broad spectrum of conditions, including asthma, inflammation, epilepsy, cardiovascular disorders, wounds, backaches, diabetes, kidney ailments, hypertension, hemorrhoids, gingivitis, shingles, stroke, and various skin conditions (20). *Cannabis* extracts have demonstrated broad-spectrum antibacterial activity (21); however, no plant-derived antibiotics, including those from *Cannabis*, have yet been successfully commercialized (22). Some laboratory studies indicate that *Cannabis* extracts are cytocompatible and can counteract the potential harm associated with individual plant components (23, 24). This effect is attributed to the presence of a wide range of bioactive compounds, particularly phytocannabinoids, including tetrahydrocannabinol, tetrahydrocannabinolic acid, cannabidiol, and cannabigerol, which act synergistically. These compounds have also been shown to exhibit selective cytotoxicity against various cancer cell lines while protecting healthy tissue from apoptosis (25). Studies further demonstrate that *Cannabis* extracts rich in tetrahydrocannabinolic acid (THCA) exhibit selective cytotoxicity against colorectal cancer (CRC) cells, such as the HT-29 cell line, while protecting healthy colon cells (26, 27). However, it is important to note that the cytotoxic effects of THCA are often observed as part of synergistic interactions with other cannabinoids and compounds present in *Cannabis* extracts. While THCA and other cannabinoids induce selective cytotoxicity against CRC cells, the anti-infective properties of *Cannabis* also extend to non-lethal mechanisms, such as disrupting biofilm formation and inhibiting QS. By targeting these less-exploited strategies, novel treatments may be developed that eradicate bacterial infections without contributing to the growing problem of antibiotic resistance. Despite these promising findings, further empirical research on *Cannabis* extracts is required due to limited pharmacological predictability, variability in extraction methods and product composition, and regulatory and methodological constraints. Therefore, this study aimed to assess the antibacterial, anti-biofilm, anti-quorum-sensing, and cytotoxic activities of *Cannabis* “Gorilla Glue 1” (GG1) against fish pathogens, particularly *A. hydrophila*, *E. tarda*, and *P. fluorescens*.

## Materials and methods

### Study site and plant material

The pot experiment was conducted in the greenhouse with minimum and maximum air temperatures ranging from 7.4 to 44.9 °C, situated at the University of South Africa, Florida Science Campus, Roodepoort, at a latitude of −26°9′29.274″ and a longitude of 27°55′17.663″ (28). The average relative humidity inside the greenhouse was maintained at 68%. Certified seeds of *Cannabis* were obtained from Marijuana SA (Pty) Ltd. (2019/092707/07) and planted in white disposable foam cups filled with the Hygro-Mix. The seeds germinated on day 10 after planting.

### Experimental design and layout

The plastic pots were arranged in a completely randomized design (CRD) consisting of 12 treatments, replicated three times. The treatment combinations consisted of N (0.21; 0.36; 0.54 g L^−1^), P (0.06; 0.12; 0.24 g L^−1^), and K (0.36; 0.6; 0.84 g L^−1^). Fertilizers were applied at week 2 after transplanting. Fertilizers used as sole sources of nitrogen, phosphorus, and potassium were urea [CO(NH_2_)2: 46% of nitrogen]; calcium superphosphate [Ca(H_2_PO_4_)2: 20%], and potassium sulfate (K_2_SO_4_: 60%), respectively. All pots were uniformly irrigated with tap water every second day using Addis’s watering can. The experiment was terminated at week 13 after planting, at the flowering stage.

### Plant collection and preparation

*Cannabis* plants treated with different levels of NPK fertilizers were harvested for leaves and cleaned at the flowering stage. The leaves were air-dried in the Biochemistry Laboratory, Florida Campus, UNISA, until completely dry, and then milled and sieved to obtain a fine powder. Leaf extracts from different treatments were prepared in acetone at a concentration of 10 mg/mL, while the working concentration of the positive control was 2 mg/mL.

### Microbial strains

The antibacterial activity of the acetone crude extracts of leaf powder was determined using the two-fold serial microdilution method. Bacterial strains of *A. hydrophila* (ATCC 35654), *E. tarda* (ATCC 15947), and *P. fluorescens* (ATCC 13525) (Analytical Technology, South Africa) were used for the antibacterial assay. The strains were maintained in brain heart infusion agar.

### *In vitro* antimicrobial serial microdilution assay

The antibacterial assay was performed using a microplate serial dilution method (29). Bacterial cultures grown overnight in brain heart infusion medium (Sigma-Aldrich, South Africa) were adjusted to McFarland standard No. 1 (equivalent to 3 × 10^8^ CFU/mL). A 100-μL aliquot of sterile distilled water was added to all wells of a 96-well microtiter plate. Prepared extracts (10 mg/mL stock concentration) were added to the first row of the microplate and serially diluted in a 1:1 ratio. One hundred microliters of the adjusted bacterial culture was then added to each well. The bacterial inoculum was exposed to final extract concentrations ranging from 2.5 to 0.01 mg/mL. Acetone and gentamicin (2 mg/mL) served as negative and positive controls, respectively. The inoculated plates were incubated at 37 °C for 18–24 h. Following incubation, 40 μL (0.2 mg/mL) of *p*-iodonitrotetrazolium violet (INT) was added to each well and incubated for 1 h. The MIC was defined as the lowest extract concentration showing growth inhibition, indicated by a decrease in red color resulting from reduced INT conversion by actively respiring bacteria. The most active extract treatments were selected for further screening.

### Anti-biofilm assay

#### Inhibition of biofilm formation

The modified protocol of (30, 31) was used to investigate the ability of the acetone leaf extracts from different treatments to prevent bacterial attachment and biofilm formation. Two stages of biofilm development were evaluated: prevention of biofilm attachment (T_0_) and eradication of a 24-h pre-formed biofilm (T_24_). For the T_0_ assay, plant extracts were added before biofilm formation, whereas for T_24_, biofilms were pre-formed for 24 h before treatment. Plant extracts were added at a final concentration of 1 mg/mL in both assays. For the T_0_ experiment, 100 μL of standardized bacterial culture (OD_590_ = 0.02, equivalent to 1.0 × 10^6^ CFU/mL) prepared in tryptone soy broth (TSB) was inoculated into sterile, flat-bottomed 96-well microtiter plates, followed by the addition of 100 μL of the plant extract. Plates were incubated at 37 °C for 24 h without shaking. For the T_24_ assay, 100 μL of standardized bacterial culture was first incubated for 24 h to allow biofilm formation before adding plant extracts. In both T_0_ and T_24_ experiments, the following controls were included: negative control (culture + TSB), positive control (culture + TSB + antibiotics: gentamicin and ciprofloxacin), sample control (sample + TSB), antibiotic control (antibiotic + TSB), and media control (TSB only). After 24 h of incubation, biofilm biomass was quantified using the modified crystal violet staining (CVS) assay.

#### Crystal violet staining assay

After incubation, the contents of the wells were removed, and the plates were washed three times with sterile distilled water to remove unattached or loosely attached cells. The plates were then air-dried and oven-dried at 60 °C for 45 min. To fix adherent cells, 150 μL of 96% methanol was added to each well for 15–20 min. Methanol was removed, and wells were stained with 100 μL of 0.1% crystal violet solution for 20 min at room temperature. Excess stain was gently removed by washing the plates at least five times with sterile distilled water. To semi-quantitatively determine biofilm biomass, the bound crystal violet was resolubilized by adding 150 μL of 100% ethanol to each well to destain the adherent cells. The plates were gently shaken, and the absorbance of each well was measured at 590 nm using a microplate reader (Epoch™ Microplate Spectrophotometer). The mean absorbance (OD_590_ nm) for each sample was calculated, and the results were expressed as percentage inhibition using the following equation:


$$\text{Percentage inhibition}\;(\%)=[\begin{array}{l} \left(\begin{array}{l} \text{ODNegative control}\\ -\text{ODSample} \end{array}\right)\\ /\text{ODNegative control} \end{array}]\times 100$$

Biofilm inhibition values ranged from 0 to 100%. Values below 0% were categorized as biofilm growth enhancement, values between 0 and 50% indicated weak anti-biofilm activity, and values above 50% represented good biofilm inhibition.

### Anti-quorum sensing

#### Inoculum preparation

A single colony of the pigment-producing bacterial strain *Chromobacterium violaceum* (ATCC 12472) was picked from an agar plate and inoculated into 10 mL of Luria–Bertani (LB) broth, followed by overnight incubation in a shaker incubator (140 rpm) at 30 °C for 24 h prior to each experiment. The working bacterial suspension was prepared by diluting the overnight-grown culture with LB broth to obtain an absorbance of 0.1 ± 0.02 at a wavelength of 590 nm, corresponding to McFarland standard No. 1 (3 × 10^8^ CFU/mL).

#### Quantitative detection of violacein inhibition in the presence of plant extracts

The anti-quorum-sensing activity of the extracts was evaluated using 48-well microplates following the protocol described by (32) with slight modifications. The bacterial suspension was prepared by inoculating a single colony of *C. violaceum* from an agar plate into 10 mL of LB broth, followed by incubation in an orbital shaker (140 rpm) at 30 °C for 24 h before each experiment. Inhibition of violacein production was achieved by transferring 1 mL of the overnight-grown *C. violaceum* into a 200-mL sterile flask and diluting it with 100 mL of LB broth. The culture was standardized to approximately 3 × 10^8^ CFU/mL by measuring absorbance at 590 nm and comparing it with McFarland standard No. 1. Subsequently, 0.5 mL of LB broth was transferred into each well of a 48-well plate, followed by the addition of 0.5 mL of extracts (10 mg/mL) and positive controls (gentamicin, ciprofloxacin, and amphotericin B at 1 mg/mL each) to their respective wells to obtain final concentrations ranging from 1.25 to 0.01 mg/mL, except for wells designated as blanks (culture and media). Then, 0.5 mL of the standardized overnight culture was added to each well, after which the plates were sealed with parafilm and incubated in an orbital shaker (140 rpm) at 30 °C for 24 h. The MIC values were interpreted as the minimum concentration of extracts and controls showing clear wells (no growth and no purple pigmentation), while the minimum quorum-sensing inhibitory concentration (MQSIC) was measured as the presence of growth (turbidity) with no purple pigmentation.

#### Violacein quantification

The anti-quorum-sensing activity of the extracts was determined according to (32) with slight modifications. The production of violacein by *C. violaceum* was measured to evaluate the anti-quorum-sensing potential of the extracts. After measuring the violacein inhibition, the plates were sealed and centrifuged at 4,000 rpm for 20 min to separate the bacteria from the culture medium. The supernatant was discarded, and the bacterial pellet was resuspended in 1 mL of 100% dimethyl sulfoxide (DMSO) and shaken in a shaker for 10 min. The supernatant (200 μL) was transferred into wells of a 96-well round-bottomed microplate in triplicate, and absorbance was measured at 595 nm. The percentage violacein inhibition was calculated using the following formula:


$$\%\text{Violacein Inhibition}={\mathrm{OD}}_{\text{control}-}{\mathrm{OD}}_{\text{test}}/{\mathrm{OD}}_{\text{control}}\times 100$$

The extract concentrations at which 50% of violacein production was inhibited (IC50) were obtained using linear regression between the percentage of violacein inhibition and the corresponding concentrations.

#### *In vitro* cytotoxicity assay

In general, most drugs or substances are metabolized in the liver and excreted by the kidneys. Therefore, Vero African green monkey kidney cells were selected to represent one of these organs. The cytotoxicity of crude acetone extracts from *Cannabis* plants was determined using a tetrazolium-based colorimetric assay, 3-(4,5-dimethylthiazol)-2,5-diphenyl tetrazolium bromide (MTT assay), as described by Mosmann (33) and slightly modified by Mármol et al. (26). Vero African green monkey kidney cells were obtained from the tissue culture laboratory of the Department of Paraclinical Sciences, Faculty of Veterinary Science, University of Pretoria. Cells were cultured in Minimum Essential Medium Eagle with L-glutamine (EMEM) (Sigma-Aldrich, USA) supplemented with 1% penicillin–streptomycin and 10% fetal bovine serum (FBS). For the experiment, EMEM supplemented with 5% FBS and 0.1% gentamicin (Genta50) (Virbac, Centurion, South Africa) was used. A 5-day-old confluent culture in 75 cm^2^ flasks was harvested and centrifuged at 910 rpm for 7 min, and the pellet was resuspended in growth medium to a seeding density of 0.1 × 10^6^ cells/mL. A sterile 96-well microplate was used, and cell suspension (100 μL) was added to each well of columns 2–11. Columns 1 and 12 were used as blanks with 200 μL of growth medium to minimize the “edge effect” and maintain humidity. The plates were incubated for 24 h at 37 °C in a 5% CO_2_ incubator until the cells reached the exponential growth phase and attached. The cells were then exposed to different concentrations of the extract samples, with doxorubicin hydrochloride (Adriblastina CSV, Pfizer, Johannesburg, South Africa) as the positive control and acetone as the negative control. From a stock concentration of 100 mg/mL of the extract sample, working concentrations were prepared using the growth medium as a diluent: 1, 0.75, 0.5, 0.25, 0.1, 0.075, 0.05, and 0.025 mg/mL. The microplates were then incubated for 48 h. After incubation, the medium was removed from the wells using a multichannel pipette attached to a pump via a plastic tube. The cells were rinsed two times with 200 μL phosphate-buffered saline (PBS, Sigma-Aldrich, Johannesburg, South Africa) and replaced with 200 μL of fresh medium. Then, 40 μL of a 5-mg/mL MTT solution of 3-(4,5-dimethylthiazol)-2,5-diphenyl tetrazolium bromide (Sigma-Aldrich, Johannesburg, South Africa) in PBS was added to each well. The plates were incubated for a further 4 h at 37 °C in the CO_2_ incubator. After incubation, the medium was carefully removed using the same multichannel pipette connected to a pump without disturbing the MTT formazan crystals in each well. Then, 100 μL of dimethyl sulfoxide (DMSO) was added to each well to dissolve the MTT crystals. Absorbance was measured immediately at 570 nm using a microplate reader (BioTek Synergy HT, Analytical and Diagnostic Products, Johannesburg, South Africa). Columns 1 and 12 were used as blanks. All concentrations were tested in triplicate, and the assay was repeated at least three times. The LC_50_ values were calculated as the concentration of plant extracts resulting in 50% cell viability compared with untreated cells. The selectivity index (SI) was calculated as LC_50_/MIC.

### Statistical analysis

Data obtained from the antimicrobial and cytotoxicity experiments were expressed as mean + standard deviation (SD), as experiments were performed in triplicate and repeated three times.

## Results

### Antibacterial activity

Generally, among the solvent extracts of different treatments, 0 g N; 0 g P; 0.84 g K exhibited the best average antibacterial activity of 0.15 against all tested pathogens (Table 1A). In contrast, 0 g N; 0 g P; 0 g K showed the weakest average MIC value (1.77) across all tested organisms (Table 1B). Solvent extract treatment 0.36 g N; 0.12 g P; 0 g K exhibited the best MIC value (0.02 mg/mL) against *E. tarda and P. fluorescens* (Table 1A). Solvent extract treatment 0 g N; 0.36 g P; 0.6 g K displayed good antibacterial activity against *E. tarda and P. fluorescens*, with MIC values of 0.07 mg/mL and 0.05 mg/mL, respectively, while 0 g N; 0 g P; 0.84 g K demonstrated excellent antibacterial activity with an MIC value of 0.07 mg/mL against both pathogens (Table 1A). Treatment 0 g N; 0.24 g P; 0 g K also showed good antibacterial activity against *E. tarda* (MIC = 0.07 mg/mL) and moderate antibacterial activity against *A. hydrophila* and *P. fluorescens*, with MIC values of 0.31 mg/mL and 0.15 mg/mL, respectively (Table 1A). Furthermore, 0.54 g N; 0 g P; 0 g K, 0.36 g N; 0.06 g P; 0.12 g K, 0.36 g N; 0.12 g P; 0.36 g K, 0.21 g N; 0.06 g P; 0.36 g K, and 0 g N; 0 g P; 0 g K displayed moderate antibacterial activity against *E. tarda* with MIC values of 0.31, 0.15, 0.15, 0.31, and 0.31 mg/mL, respectively (Tables 1B, C).

**Table 1a:** Antibacterial activity of acetone extracts of *Cannabis* (GG1) against selected fish pathogens.

Minimum inhibitory concentration [MIC (mg/mL)]						
Organisms	Plant extracts				Controls	
	0.36 g K; 0.12 g P; 0 g K	0 g N; 0.36 g P; 0.6 g K	0 g N; 0 g P; 0.84 g K	0 g N; 0.24 g P; 0 g K	Gentamicin	Acetone
*Aeromonas hydrophila*	1.25	0.62*	0.31*	0.31*	0.06	>2.50
*Edwardsiella tarda*	**0.02**	**0.07**	**0.07**	**0.07**	0.06	>2.50
*Pseudomonas fluorescens*	**0.02**	**0.05**	**0.07**	0.15*	0.06	>2.50
Average	0.43*	0.74	0.15*	0.18*	0.06	>2.50

Bold values indicate MIC ≤0.1 mg/mL (significantly active), 0.1 < MIC ≤ 0.625 mg/mL (moderately active), and MIC >0.625 mg/mL (weak). * = moderate activity.

**Table 1B:** Antibacterial activity of acetone extracts of *Cannabis* (GG1) against selected fish pathogens.

Minimum inhibitory concentration [MIC (mg/mL)]						
Organisms	Plant extracts				Controls	
	0.36 g N; 0 g P; 0.36 g K	0.21 g N; 0.12 g P; 0.36 g K	0.21 g N; 0.06 g P; 0.36 g K	0 g N; 0 g P; 0 g K	Gentamicin	Acetone
*Aeromonas hydrophila*	>2.5 0	>2.50	1.25	>2.50	0.06	>2.50
*Edwardsiella tarda*	0.62*	0.62*	0.31*	0.31*	0.06	>2.50
*Pseudomonas fluorescens*	1.25	0.62*	0.62*	2.50	0.06	>2.50
Average	1.46	1.25	0.73	1.77	0.06	>2.50

Bold values indicate MIC ≤0.1 mg/mL (significantly active), 0.1 < MIC ≤ 0.625 mg/mL (moderately active), and MIC >0.625 mg/mL (weak). * = moderate activity.

**Table 1C:** Antibacterial activity of acetone extracts of *Cannabis* (GG1) against selected fish pathogens.

Minimum inhibitory concentration [MIC (mg/mL)]						
Organisms	Plant extracts				Controls	
	0.36 g N; 0.12 g P; 0.6 g K	0.54 g N; 0 g P; 0 g K	0.36 g N; 0.06 g P; 0.12 g K	0.36 g N; 0.12 g P; 0.36 g K	Gentamicin	Acetone
*Aeromonas hydrophila*	0.23*	2.50	2.50	>2.50	0.06	>2.50
*Edwardsiella tarda*	>2.50	0.31*	0.15*	0.15*	0.06	>2.50
*Pseudomonas fluorescens*	0.62*	0.62	0.94	2.50	0.06	>2.50
Average	1.12	1.14	1.20	1.72	0.06	>2.50

Bold values indicate MIC ≤0.1 mg/mL (significantly active), 0.1 < MIC ≤ 0.625 mg/mL (moderately active), and MIC >0.625 mg/mL (weak). * = moderate activity.

### Plant extract yields

Dried *Cannabis* leaves collected after exposure to different fertilizing regimens were extracted using acetone in this study. The highest percentage yield (13.73%) was obtained from solvent extract treatment 0.54 g N; 0 g P; 0 g K, followed by 0.36 g N; 0.12 g P; 0.6 g K, and 0.36 g N; 0.06 g P; 0.12 g K, which yielded 11.06 and 10.92%, respectively (Tables 2A–C). The lowest percentage yield (0.76%) was obtained from the solvent extract treatment 0 g N; 0 g P; 0 g K (control) (Table 2C). The extraction yield and bioactivities of extracts using different extractants vary considerably (34), hence the choice of acetone in this study. The average total antibacterial activity (TAA) values of the plant extracts ranged from 11 to 3,199.12 mL/g against all tested bacteria (Tables 2A–C, 3). The highest TAA value (3,199.12 mL/g; Table 2A) was produced by solvent extract treatment 0.36 g N; 0.12 g P; 0 g K against *E. tarda* and *P. fluorescens*.

**Table 2A:** Percentage yields and total antibacterial activity (TAA) of acetone extracts of *Cannabis* (GG1) against selected fish pathogens.

Total activity (mL/g)				
Organisms	Plant extracts			
	0.36 g N; 0.12 g P; 0 g K	0 g N; 0.36 g P; 0.6 g K	0 g N; 0 g P; 0.84 g K	0 g N; 0.24 g P; 0 g K
*Aeromonas hydrophila*	51.191	102.44	222.54	92.57
*Edwardsiella tarda*	3,199.12	907.00	985.00	409.00
*Pseudomonas fluorescens*	3,199.12	1,270.00	985.00	191.00
% Yield	7.83%	8.19%	8.86%	2.66%
Average	2,149.81	759.81	730.86	230.86

**Table 2B:** Percentage yields and total antibacterial activity (TAA) of acetone extracts of *Cannabis* (GG1) against selected fish pathogens.

Total activity (mL/g)				
Organisms	Plant extracts			
	0.36 g N; 0.12 g P; 0.6 g K	0.54 g N; 0 g P; 0 g K	0.36 g N; 0.06 g P; 0.6 g K	0.36 g N; 0.12 g P; 0.36 g K
*Aeromonas hydrophila*	388.07	45.52	36.13	2,907.02
*Edwardsiella tarda*	35.00	367.00	602.00	522.00
*Pseudomonas fluorescens*	1,496.10	183.00	96.00	31.00
% Yield	11.06%	13.73%	10.92%	9.00%
Average	639.72	198.51	244.68	1,153.34

**Table 2C:** Percentage yields and total antibacterial activity (TAA) of acetone extracts of *Cannabis* (GG1) against selected fish pathogens.

Total activity (mL/g)				
Organisms	Plant extracts			
	0.36 g N; 0 g P; 0.36 g K	0.21 g N; 0.12 g P; 0.36 g K	0.21 g N; 0.06 g P; 0.36 g K	0 g N; 0 g P; 0 g K
*Aeromonas hydrophila*	38.26	32.95	66.40	111.89
*Edwardsiella tarda*	154.00	132.00	269.00	90.00
*Pseudomonas fluorescens*	76.00	132.00	133.00	11.00
% Yield	8.70%	8.39%	9.94%	0.76%
Average	89.42	98.98	156.13	70.96

**Table 3:** Anti-biofilm potential of the selected *Cannabis* extracts against fish pathogens.

S/N	Plant extracts	% Inhibition					
		*Aeromonas hydrophila*		*Edwardsiella tarda*		*Pseudomonas fluorescens*	
		T_0_	T_24_	T_0_	T_24_	T_0_	T_24_
1	0.36 g N; 0.12 g P; 0 g K	−	−	−	++	+	++
2	0 g N; 0.36 g P; 0.6 g K	−	++	++	++	−	−
3	0 g N; 0 g P; 0.84 g K	++	−	−	++	−	++
4	0 g N; 0.24 g P; 0 g K	++	+	−	++	−	++
5	0.36 g N; 0.12 g P; 0.6 g K	++	++	−	++	++	++
6	0.54 g N; 0 g P; 0 g K	++	++	−	++	++	++
7	0.36 g N; 0.06 g P; 0.12 g K	++	++	+	++	++	+
8	0.36 g N; 0.12 g P; 0.36 g K	++	++	+	++	++	−
9	0.36 g N; 0 g P; 0.36 g K	++	++	+	++	++	−
10	0.21 g N; 0.12 g P; 0.36 g K	++	−	−	++	++	−
11	0.21 g N; 0.06 g P; 0.36 g K	++	+	++	++	++	−
12	0 g N; 0 g P; 0 g K	++	−	++	++	++	−
13	Gentamicin	+	−	−	+	−	++
14	Ciprofloxacin	++	−	++	+	++	++

NB: Good (++) ABF activity (>50% inhibition); poor (+) ABF activity (0–50% inhibition); no (−) ABF activity (≤0%).

### Anti-biofilm activity

The results of the anti-biofilm (ABF) potential of the *Cannabis* acetone extracts against selected fish pathogens are presented in Table 3. Extracts or fractions resulting in inhibition above 50% were considered to exhibit good ABF activity (++), while those with inhibition between 0 and 50% were regarded as having poor ABF activity (+), and values <0% (−) were considered to indicate no inhibition or enhancement of biofilm development and growth. All the tested extracts showed the capacity to either prevent or disrupt formed biofilms, while solvent extract treatment 0.36 g N; 0.06 g P; 0.12 g K displayed the best ABF activity. The results demonstrated that several extracts exhibited both preventive and disruptive effects against the tested pathogens. Specifically, solvent extract treatments 0 g N; 0.24 g P; 0 g K, 0.54 g N; 0 g P; 0 g K, 0.36 g N; 0.06 g P; 0.12 g K, 0.36 g N; 0.12 g P; 0.36 g K, and 0.36 g N; 0 g P; 0.36 g K were effective in both preventing and destroying (>50% inhibition) *A. hydrophila*. In addition, a broader range of treatments—including 0 g N; 0 g P; 0.84 g K, 0 g N; 0.24 g P; 0 g K, 0.36 g N; 0.12 g P; 0.6 g K, 0.54 g N; 0 g P; 0 g K, 0.36 g N; 0.06 g P; 0.12 g K, 0.36 g N; 0.12 g P; 0.36 g K, 0.36 g N; 0 g P; 0.36 g K, 0.21 g N; 0.12 g P; 0.36 g K, 0.21 g N; 0.06 g P; 0.36 g K, and 0 g N; 0 g P; 0 g K—showed preventive activity against the same pathogen. Solvent extract treatments 0 g N; 0.36 g P; 0.6 g K, 0.21 g N; 0.06 g P; 0.36 g K, and 0 g N; 0 g P; 0 g K were capable of both preventing and destroying (>50% inhibition) *E. tarda*. In the case of *P. fluorescens*, treatments 0.36 g N; 0.12 g P; 0.6 g K and 0.54 g N; 0 g P; 0 g K demonstrated both preventive and disruptive effects (>50% inhibition), whereas treatments 0.36 g N; 0.12 g P; 0.6 g K, 0.54 g N; 0 g P; 0 g K, 0.36 g N; 0.06 g P; 0.12 g K, 0.36 g N; 0.12 g P; 0.36 g K, 0.36 g N; 0 g P; 0.36 g K, 0.21 g N; 0.12 g P; 0.36 g K, 0.21 g N; 0.06 g P; 0.36 g K, and 0 g N; 0 g P; 0 g K were effective in preventing its growth. These findings highlight the broad-spectrum potential of some extracts, particularly treatments 0.54 g N; 0 g P; 0 g K, 0.21 g N; 0.06 g P; 0.36 g K, and 0 g N; 0 g P; 0 g K, which showed activity across multiple pathogens. Furthermore, inhibition of biofilm formation at T_0_ against *A. hydrophila and P. fluorescens* was higher (>50% inhibition) than at T_24_. In contrast, inhibition at T_0_ against *E. tarda* was lower (0–50% inhibition) than that observed at T_24_.

### Anti-quorum-sensing activity

Interference of the *Cannabis* acetone extracts with QS-mediated purple pigment production in *C. violaceum* is indicative of QS inhibition. The results in Table 4 indicate that the solvent extracts of different treatments exhibited anti-quorum-sensing (AQS) activity against *C. violaceum*. All solvent extract treatments showed good anti-quorum-sensing activity, while treatment 0.36 g N; 0.06 g P; 0.12 g K achieved the highest violacein inhibition (98.61%) at a concentration of 1.25 mg/mL, followed by 0.36 g N; 0 g P; 0.36 g K, which demonstrated 97.85% inhibition at the same concentration.

**Table 4:** Anti-quorum-sensing activity and minimum inhibitory concentrations (MICs) of selected extracts of *Cannabis* (GG1) against *C. violaceum* (ATCC 12472).

S/N	Plant extracts	Violacein inhibition (%)								MIC (mg/mL)	MQSIC (mg/mL)
		Concentrations (mg/mL)									
		0.01	0.02	0.04	0.08	0.16	0.31	0.63	1.25		
1	0.36 g N; 0.12 g P; 0 g K	91.90	66.71	92.15	96.22	71.08	94.84	92.53	94.55	1.25	0.63
2	0 g N; 0.36 g P; 0.6 g K	92.80	66.58	91.29	96.48	69.68	93.09	90.99	97.44	1.25	0.63
3	0 g N; 0 g P; 0.84 g K	93.72	65.99	91.79	95.27	71.24	91.44	88.90	94.80	1.25	0.63
4	0 g N; 0.24 g P; 0 g K	91.13	66.67	89.31	91.19	69.60	93.17	87.68	93.28	>1.25	1.25
5	0.36 g N; 0.12 g P; 0.6 g K	92.95	66.50	91.49	91.45	67.17	93.52	90.05	95.38	1.25	0.63
6	0.54 g N; 0 g P; 0 g K	92.10	61.78	91.82	94.60	67.46	94.03	90.28	93.12	1.25	0.63
7	0.36 g N; 0.06 g P; 0.12 g K	92.90	66.75	92.62	94.50	66.88	92.81	91.06	98.61	1.25	0.63
8	0.36 g N; 0.12 g P; 0.36 g K	92.90	61.52	87.62	94.69	64.99	92.81	89.54	95.22	1.25	0.63
9	0.36 g N; 0 g P; 0.36 g K	92.90	61.99	92.62	92.59	70.34	93.90	90.69	97.85	1.25	0.63
10	0.21 g N; 0.12 g P; 0.36 g K	91.36	65.22	90.82	96.32	69.72	94.23	91.67	97.47	1.25	0.63
11	0.21 g N; 0.06 g P; 0.36 g K	92.26	66.12	92.43	96.06	70.13	93.57	89.00	96.58	<0.01	<0.01
12	0 g N; 0 g P; 0 g K	93.21	65.48	94.22	97.66	72.48	94.18	87.95	92.42	1.25	0.63
13	Gentamicin	92.21	65.48	92.29	93.90	66.96	92.08	87.99	93.57	<0.01	<0.01
14	Ciprofloxacin	92.54	65.48	92.65	96.19	69.84	93.27	89.51	95.69	<0.01	<0.01
15	Amphotericin B	63.39	94.61	94.04	78.37	93.07	91.20	86.09	92.98	0.63	0.31

Bold values indicate MIC ≤0.1 mg/mL (significantly active), 0.1 < MIC ≤ 0.625 mg/mL (moderately active), and MIC >0.625 mg/mL (weak). MQSIC, minimum quorum-sensing inhibitory concentration; MIC, minimum inhibitory concentration.

### Cytotoxicity and selectivity index (SI)

The cytotoxicity activity against Vero kidney cells and the selectivity indices of solvent extracts from different fertilizer treatments are presented in Table 5. Solvent extract 0 g N; 0.24 g P; 0 g K was the most toxic, with an LC_50_ value of 0.04 mg/mL against Vero cells, while solvent extracts 0.36 g N; 0.12 g P; 0 g K, 0 g N; 0.36 g P; 0.6 g K, and 0 g N; 0 g P; 0 g K showed moderate toxicity with LC_50_ values of 0.06 mg/mL. The LC_50_ values for the other solvent extracts were all greater than 0.1 mg/mL, indicating that they are non-cytotoxic to mammalian (Vero) cells. Specifically, solvent extracts 0 g N; 0 g P; 0.84 g K, 0.54 g N; 0 g P; 0 g K, and 0.36 g N; 0.06 g P; 0.12 g K showed LC_50_ values of 0.10 mg/mL, followed by 0.36 g N; 0.12 g P; 0.6 g K, 0.36 g N; 0 g P; 0.36 g K, and 0.21 g N; 0.06 g P; 0.36 g K with LC_50_ values of 0.09 mg/mL. Solvent extract 0.21 g N; 0.12 g P; 0.36 g K had an LC_50_ value of 0.08 mg/mL, while solvent extract 0.36 g N; 0.12 g P; 0.36 g K had an LC_50_ value of 0.07 mg/mL. Cytotoxicity (mg/mL) and MIC (mg/mL) values were used to calculate the selectivity index (SI) of plant extracts (SI = LD_50_/MIC), which represents the safety margin of the extract (35). In this study, solvent extract 0.36 g N; 0.12 g P; 0 g K displayed the highest SI (3.00) against *E. tarda* and *P. fluorescens*, followed by 0 g N; 0 g P; 0.84 g K with an SI of 2.29 against the same pathogens. Solvent extract 0 g N; 0.36 g P; 0.6 g K showed a good SI (1.20) only against *P. fluorescens*.

**Table 5:** Cytotoxicity (LC_50_ in mg/mL) and selectivity index (SI) of acetone extracts of *Cannabis* (GG1) against Vero monkey kidney cells.

S/N	Extracts	LC_50_ (mg/mL)	Selectivity index			
			Vero cells			
			Organisms			
			*A. hydrophila*	*E. tarda*	*P. fluorescens*	Average
1	0.36 g N; 0.12 g P; 0 g K	0.06 ± 0.01	0.04	**3.00**	**3.00**	**2.01**
2	0 g N; 0.36 g P; 0.6 g K	0.06 ± 0.01	0.09	0.85	**1.20**	0.70
3	0 g N; 0 g P; 0.84 g K	0.16 ± 0.05	0.51	**2.29**	**2.29**	**1.70**
4	0 g N; 0.24 g P; 0 g K	**0.04 ± 0.01**	0.13	0.57	0.27	0.32
5	0.36 g N; 0.12 g P; 0.6 g K	0.09 ± 0.04	0.39	0.04	0.15	0.20
6	0.54 g N; 0 g P; 0 g K	0.10 ± 0.03	0.04	0.32	0.16	0.17
7	0.36 g N; 0.06 g P; 0.12 g K	0.10 ± 0.05	0.04	0.67	0.12	0.28
8	0.36 g N; 0.12 g P; 0.36 g K	0.07 ± 0.03	0.02	0.47	0.03	0.17
9	0.36 g N; 0 g P; 0.36 g K	0.09 ± 0.03	0.04	0.15	0.07	0.09
10	0.21 g N; 0.12 g P; 0.36 g K	0.08 ± 0.03	0.03	0.13	0.13	0.10
11	0.21 g N; 0.06 g P; 0.36 g K	0.09 ± 0.04	0.07	0.10	0.15	0.11
12	0 g N; 0 g P; 0 g K	0.06 ± 0.02	0.02	0.19	0.02	0.08
13	Doxorubicin	0.00562 ± 0.00007	–	–	–	

LC_50_ calculated in mg/ml; the Bold values under LC_50_ represent the toxic concentration of solvent extract to the Vero cells, while bold values under the selectivity index >1 are more toxic to bacterial pathogens.

## Discussion

### Antibacterial activity

For decades, *Cannabis* plants have been used to treat infections, and currently, novel *Cannabis*-derived antibiotics are highly promising and are progressing through clinical trials (36), due to their established medicinal properties (37, 38). Solvent extract treatments with MIC values ≤0.1 mg/mL were considered to exhibit significant activity, while moderate activity was defined as MIC values between 0.1 and 0.625 mg/mL, and MIC values >0.625 mg/mL were regarded as weak or poor activity (39). The antibacterial activity of *Cannabis* extract observed in this study is consistent with findings from other medicinal plant extracts with promising antibacterial activities. In agreement with the present results, (40) reported potent antibacterial activity of acetone extracts of *Azadirachta indica* against *Pseudomonas* species, with the lowest MIC value of 0.18 mg/mL. The results obtained by (41) demonstrated that aqueous extracts of *Terminalia arjuna* showed strong antibacterial activity against *P. fluorescens* at a concentration of 25 μL. Another study reported that methanolic leaf extracts of *Ziziphus mauritiana* exhibited high antibacterial activity against *P. fluorescens* at concentrations of 25 and 50 μg/μL (42). Generally, there is limited information on the antibacterial activity of medicinal plants against *E. tarda*. However, (43) reported that extracts of *Moringa oleifera* exhibited strong antibacterial activity against *E. tarda* at a concentration of 375 mg/L. The present findings are also consistent with those reported by (44), who showed that coffee leaf extracts exhibited strong antibacterial activity against *E. tarda* at concentrations ranging from 20 to 80%. Furthermore, leaf extracts of *Lantana camara* have been reported to inhibit the growth of *E. tarda* at a concentration of 200 mg/L (45). Based on our findings, solvent extract treatment 0 g N; 0.24 g P; 0 g K exhibited moderate antibacterial activity against *A. hydrophila* with an MIC value of 0.31 mg/mL, which contrasts with the findings of (46), where leaf extracts of *Nelumbo nucifera* showed potent activity against *A. hydrophila* with an MIC value of 31.25 μL/mL using an ethanol–water solvent system. In addition, (47) demonstrated that guava leaf extracts inhibited the growth of *A. hydrophila* at a concentration of 50%. Varying NPK fertilizer concentrations have been shown to influence plant antibacterial activity by affecting the biosynthesis of antibacterial secondary metabolites, such as flavonoids and phenolics, which possess antimicrobial properties (48). In this study, solvent extracts obtained under different fertilizer treatments, particularly 0.36 g N; 0.12 g P; 0 g K, 0 g N; 0.36 g P; 0.6 g K, 0 g N; 0.24 g P; 0 g K, and 0 g N; 0 g P; 0.84 g K, appeared to enhance the production of these bioactive compounds, resulting in strong antibacterial activity against *E. tarda and P. fluorescens*. However, bioassay-guided fractionation is necessary to isolate and identify the specific compounds responsible for these antibacterial activities. The results of this study suggest that *Cannabis* extracts have promising anti-pathogenic potential and warrant further investigation. To the best of our knowledge, this is the first study to investigate the antibacterial activities of *Cannabis* solvent extracts obtained under different fertilizer treatments against fish pathogens.

### Plant extract yields

The yield of a plant extract is important for calculating total activity and comparing plants for bioprospecting (49). In this study, acetone solvents offered the best yields in most of the tested *Cannabis* extracts obtained under different fertilizer treatments; however, this does not necessarily translate into efficient extraction of antimicrobial substances. Similar to our study (22), acetone has consistently proven to be an effective extractant for screening and isolating antimicrobial compounds from plants. This is because acetone demonstrates a high capacity to extract compounds with a wide range of polarity (49). However, this does not imply that other solvents are not equally useful, as the solubility of plant extracts differs depending on the solvent and plant part used, as well as the phytochemical composition. The potency of a plant extract, referred to as TAA, can be determined on the basis of both the MIC (mg/mL) and extract yield (mg/g), which together indicate the volume (mL) to which the extract obtained from 1 g of plant material can be diluted while still inhibiting bacterial growth. In this study, solvent extract treatment 0.36 g N; 0.12 g P; 0 g K exhibited the highest mean TAA (3,199.12 mL/g) against *E. tarda* and *P. fluorescens,* suggesting that extracts obtained from 1 g of this treatment remain effective after substantial dilution and can still inhibit bacterial growth. The TAA is therefore useful for identifying suitable plant extracts for compound isolation and bioprospecting (34). It is worth noting that the antibacterial efficiency of plant extracts may be attributed to continuous plant defense against phytopathogens in their environment, which stimulates the production of broad-spectrum antimicrobial compounds (50).

### Anti-biofilm activity

Detecting and diagnosing biofilm-related infections pose significant hurdles due to their resilient nature, since mature biofilms intensify the development of antimicrobial resistance. Consequently, the efficacy of previously active antibiotics against planktonic cells becomes weakened. According to the previous study, the eradication of more than 50% of the pre-formed biofilm was considered indicative of notable anti-biofilm activity (49). Based on our findings, many of these *Cannabis* acetone leaf extracts showed the ability to inhibit or disrupt formed biofilms against the selected bacterial pathogens; however, few reports have explored their ability to interfere with biofilm formation and QS signaling mechanisms. The results obtained in this study are consistent with those reported by (51), which showed that the *Limonia acidissima* L. methanol and ethyl acetate extracts exhibited pronounced biofilm inhibitory effects, with >50% inhibition against *A. hydrophila* at concentrations of 250 and 500 μg/mL. Similar observations were reported by (2), who investigated the ABF activity of tea tree and peppermint essential oils against *A. hydrophila* and found that both exhibited strong ABF activity at concentrations of 0.0078 and 0.015 μL/mL, respectively. In addition, our findings are supported by (52), which showed that leaf extracts of *Dendrophthoe falcata* demonstrated good ABF activity (>50% inhibition) against *A. hydrophila*. Similarly, our study demonstrated that solvent extract treatments 0 g N; 0.36 g P; 0.6 g K, 0.21 g N; 0.06 g P; 0.36 g K, and 0 g N; 0 g P; 0 g K showed good inhibitory activity (>50% inhibition) against *E. tarda*. Most studies have investigated the antibacterial activity of plant extracts against *E. tarda*; however, this appears to be the first study to determine the ABF activity of *Cannabis* against this pathogen. In the case of *P. fluorescens*, solvent extract treatments 0.36 g N; 0.12 g P; 0.6 g K and 0.54 g N; 0 g P; 0 g K revealed both preventive and disruptive effects (>50% inhibition). In addition, solvent extract treatments 0.36 g N; 0.12 g P; 0.6 g K, 0.54 g N; 0 g P; 0 g K, 0.36 g N; 0.06 g P; 0.12 g K, 0.36 g N; 0.12 g P; 0.36 g K, 0.36 g N; 0 g P; 0.36 g K, 0.21 g N; 0.12 g P; 0.36 g K, 0.21 g N; 0.06 g P; 0.36 g K, and 0 g N; 0 g P; 0 g K were effective in preventing its growth. These findings are consistent with those reported by (53), where leaf extracts of *Hibiscus sabdariffa* demonstrated strong biofilm inhibition against *Pseudomonas aeruginosa* at a concentration of 0.151 mg/mL. Another study reported that methanolic leaf extracts of *Bergenia ciliata* and ethanolic leaf extracts of *Clematis grata* efficiently inhibited biofilm formation of *P. aeruginosa*, with 81 and 80% inhibition, respectively (54). Our observations are further supported by (55), which showed that *Trigonella foenum-graceum* extracts exhibited strong biofilm inhibition against *P. aeruginosa.* These results are also consistent with those of (56), who reported that methanol extracts of *A. marina* leaves not only inhibited initial cell adhesion and biofilm formation by *P. fluorescens* but also disrupted pre-formed biofilms, with IC_50_ values of 42.0 and 45.8 mg/mL. Similarly, (57) reported the most significant anti-biofilm activity against *Salmonella typhimurium* by acetone extracts of *Vachellia xanthophloea*. Furthermore, our study showed that *A. hydrophila* and *P. fluorescens* were inhibited by most extracts at T_0,_ indicating that prevention of biofilm attachment and growth is easier to achieve than inhibition of pre-formed biofilms at T_24_. Similarly, (31) evaluated the ABF activity of alcoholic extracts of *Allium sativum* against *Escherichia coli* and observed higher inhibition values for biofilm formation than for disruption of pre-formed biofilms. Interestingly, *E. tarda* was inhibited by most extracts at T_24,_ highlighting the effectiveness of *Cannabis* acetone extracts in preventing and disrupting biofilm formation by this pathogenic bacterium.

### Anti-quorum-sensing activity

Targeting QS signaling emerges as an innovative strategy, as this mechanism focuses on virulence factors such as biofilms and others, thereby disarming pathogenic bacteria (58). The preliminary assessment of AQS activity of *Cannabis* leaf extracts was confirmed through the inhibition of violacein formation in *C. violaceum*. Violacein, a pigment produced by *C. violaceum* in response to QS signaling, serves as an indicator of quorum-sensing activity. The results demonstrated that the *Cannabis* leaf extracts exhibited anti-quorum-sensing activity against the biosensor strain *C. violaceum*. All tested solvent extract treatments showed good AQS activity against *C. violaceum,* while solvent extract treatment 0.36 g N; 0.06 g P; 0.12 g K achieved the highest violacein inhibition of 98.61% at a concentration of 1.25 mg/mL. This observation aligns with a previous study on *Terminalia catappa*, which reported effective inhibition of violacein production at a concentration of 0.0625 mg/mL *in vitro* (59). Similarly, *T. catappa*, *T. bellerica*, *T. chebula*, and *T. macroptera* have been reported to attenuate QS in *Pseudomonas aeruginosa* (16, 59, 60). The findings of this study are consistent with those of (61), in which *Artemisia argyi* leaf extracts reduced violacein formation in *C. violaceum*. Another study by (9) reported that *Melastoma candidum* leaf extracts inhibited violacein production in *C. violaceum*. Furthermore, the findings of this study are consistent with those of (62), who observed reduced violacein production (up to 38.34%) in *C. violaceum* for *Melianthus comosus*, *Plectranthus ecklonii,* and *Pelargonium sidoides* extracts. These results reinforce the notion that *Cannabis* extracts effectively inhibit quorum-sensing-regulated violacein synthesis in *C. violaceum*. Overall, the medicinal activities displayed by *Cannabis* leaf extracts against these bacterial pathogens may be attributed to the presence of biologically active compounds in acetone extracts, such as cannabinoids, phenolics, flavonoids, quinones, alkaloids, terpenoids, and polystyrenes, which play key roles in microbial pathogenicity and are involved in the inhibition of QS molecules as well as biofilm formation (63).

### Cytotoxicity and selectivity index

The widespread assumption of safety for plant extracts and other natural products is erroneous; therefore, it is essential to conduct cytotoxicity testing to provide scientific validation of their safety. According to (39), when screening plant extracts for antimicrobial potential, there is a need to evaluate their safety on mammalian cell lines. Plant extracts showing sensitivity to cell lines with LC_50_ values >0.1 mg/mL are considered non-cytotoxic, those with LC_50_ values ≥0.06 and <0.1 mg/mL are considered moderately toxic, and extracts with LC_50_ values <0.06 mg/mL are considered toxic. Solvent extracts with good antibacterial activity (from significant to moderate) were selected for cytotoxicity testing. Most solvent extracts tested were non-toxic against Vero cells, except solvent extract 0 g N; 0.24 g P; 0 g K, which was toxic to Vero cells with an LC_50_ of 0.04 mg/mL, and solvent extracts 0.36 g N; 0.12 g P; 0 g K, 0 g N; 0.36 g P; 0.6 g K, and 0 g N; 0 g P; 0 g K, which showed moderate toxicity with LC_50_ values of 0.06 mg/mL. A similar study by (49) reported that acetone crude extracts of *Eugenia umtamvunensis* and *Syzygium legatii* had non-toxic effects on Vero cells, with LC_50_ values of 0.82 and 0.14 mg/mL, respectively. In addition, (4) reported that acetone crude extracts of *Searsia leptodictya, S. lancea, S. batophylla, Bauhinia galpinii,* and *B. bowkeri* were not toxic to Vero cells, with LC_50_ values of 0.11, 0.20, 0.15, and 0.51 mg/mL, respectively. Furthermore, similar to our findings, (57) reported that the acetone extract of *Elephantorrhiza elephantina* had a high LC_50_ value of 3.6945 ± 0.1149 mg/mL against Vero cells. However, a variety of cell lines should be used to assess the safety of plant extracts, since findings from a single cell model may not apply to whole organisms, although the use of Vero cells reflects general toxicity. The selectivity index (SI) expresses the relationship between antimicrobial and cytotoxic activities of plant extracts on bacterial and normal cells, ensuring that biological activity is not attributed solely to *in vitro* cytotoxicity. According to (64), plant extracts with SI values <1 indicate that the extracts are relatively less toxic to bacteria and more toxic to mammalian cells. Thus, extracts with SI >1 may be relatively safer to use *in vivo*, as they are less toxic to mammalian cells but more toxic to pathogens. Therefore, solvent extract 0.36 g N; 0.12 g P; 0 g K exhibited the highest SI values against Vero cells and could be considered promising for further research (65). However, *in vivo* testing is necessary to validate the efficacy and safety of these *Cannabis* extracts. These results are consistent with findings reported by (66), where acetone extracts of *Carpobrotus edulis* revealed a high selectivity index of 17.07 against *Enterobacter cloacae.* Another study by (11) demonstrated excellent SI values of 25.18 and 50.75 for acetone extracts of *B. galpinii and B. bowkeri* against *Salmonella enteritidis.* These *Cannabis* extracts show potential for development into medicinal products for controlling antimicrobial infections using herbal remedies. Alternatively, isolation of active compounds may provide templates for the development of new drugs.

## Conclusion

Little was known about the different antimicrobial activities of *Cannabis* (GG1) before this study. The solvent extracts of plants grown under different fertilizer treatments had good antibacterial activity against the planktonic and sessile forms of the bacterial pathogens investigated. The different levels of NPK fertilizers enhanced the production of bioactive compounds, which are responsible for the potent antibacterial activities against these fish pathogens. This study highlighted the significance of assessing the unexplored ABF and AQS properties of *Cannabis* extracts. Numerous solvent extracts demonstrated no toxicity, positioning them as promising options for the development of herbal products or for the isolation of novel pure compounds. These pure compounds could function as templates for new antimicrobial medications. Furthermore, these solvent extracts represent a promising candidate for subsequent *in vivo* testing as phytogenic feed additives to combat devastating fish pathogens.

## Data Availability

The original contributions presented in the study are included in the article/supplementary material, further inquiries can be directed to the corresponding author.

## References

[1] FAO. The state of world fisheries and aquaculture 2022. Rome: Towards Blue Transformation (2022).

[2] Hudecová P Koščová J Hajdučková V Király J Horňak P. Antibacterial and antibiofilm activity of essential oils against *Aeromonas* spp. isolated from rainbow trout. Animals. (2024) 14:320. doi: 10.3390/ani14223202 39595255 PMC11591162

[3] Irshath AA Rajan AP Vimal S Prabhakaran VS Ganesan R. Bacterial pathogenesis in various fish diseases: recent advances and specific challenges in vaccine development. Vaccine. (2023) 11:470. doi: 10.3390/vaccines11020470 PMC9968037 36851346

[4] Novoslavskij A Terentjeva M Eizenberga I Valciņa O Bartkevičs V Bērziņš A. Major foodborne pathogens in fish and fish products: a review. Ann Microbiol. (2016) 66:1–15. doi: 10.1007/s13213-015-1102-5

[5] Hegde A Kabra S Basawa RM Khile DA Abbu RUF Thomas NA . Bacterial diseases in marine fish species: current trends and future prospects in disease management. World J Microbiol Biotechnol. (2023) 39:317. doi: 10.1007/s11274-023-03755-5 37743401 PMC10518295

[6] Chen MC Wang JP Liu B Zhu YJ Xiao R Yang WJ . Biocontrol of tomato bacterial wilt by the new strain *Bacillus velezensis* FJAT-46737 and its lipopeptides. BMC Microbiol. (2020) 20:2–12. doi: 10.1186/s12866-020-01851-2 32539679 PMC7296739

[7] Senthamarai MD Rajan MR Bharathi PV. Current risks of microbial infections in fish and their prevention methods: a review. Microb Pathog. (2023) 185:106400. doi: 10.1016/j.micpath.2023.106400 37863271

[8] Maldonado-Miranda JJ Castillo-Pérez LJ Ponce-Hernández A Carranza-Álvarez C. Summary of economic losses due to bacterial pathogens in aquaculture industry In: Bacterial fish diseases. Amsterdam, The Netherlands: Elsevier (2022). 399–417.

[9] Pangastuti A Sari SL Budiharjo A Fitri ST Sayekti P Putri SR. Screening of some Indonesian medicinal plant extracts for anti-quorum sensing activity to prevent *Aeromonas hydrophila* infection in *Oreochromis niloticus*. Biodiversitas. (2021) 22:3517–22. doi: 10.13057/biodiv/d220851

[10] Fu J Zhang Y Lin S Zhang W Shu G Lin J . Strategies for interfering with bacterial early-stage biofilms. Front Microbiol. (2021) 12:675843. doi: 10.3389/fmicb.2021.675843 34168632 PMC8217469

[11] Adeyemo RO Famuyide IM Dzoyem JP Joy ML. Anti-Biofilm, Antibacterial, and anti-quorum sensing activities of selected south African plants traditionally used to treat diarrhoea. J Evid Based Complement Alternat Med. (2022) 2022:1–12. doi: 10.1155/2022/1307801 PMC9534605 36212949

[12] Zhao A Sun J Liu Y. Understanding bacterial biofilms: from definition to treatment strategies. Front Cell Infect Microbiol. (2023) 13:1137947. doi: 10.3389/fcimb.2023.1137947 37091673 PMC10117668

[13] Dupont CA Bourigault Y Osmond T Nier M Barbey C Latour X . *Pseudomonas fluorescens* MFE01 uses 1-undecene as aerial communication molecule. Front Microbiol. (2023) 14:1264801. doi: 10.3389/fmicb.2023.1264801 37908545 PMC10614000

[14] Jamal M Ahmad W Andleeb S Jalil F Imran M Nawaz MA . Bacterial biofilm and associated infections. J Chin Med Assoc. (2018) 81:7–11. doi: 10.1016/j.jcma.2017.07.012, 29042186

[15] Sharma D Misba L Khan AU. Antibiotics versus biofilm: an emerging battleground in microbial communities. ARIC. (2019) 8:1–10. doi: 10.1186/s13756-019-0533-3 31131107 PMC6524306

[16] Ganesh SP Rai R. Attenuation of quorum-sensing-dependent virulence factors and biofilm formation by medicinal plants against antibiotic resistant *Pseudomonas aeruginosa*. J Tradit Complement Med. (2018) 8:170–7. doi: 10.1016/j.jtcme.2017.05.008 29322006 PMC5755981

[17] Nguta JM Appiah-Opong R Nyarko AK Yeboah-Manu D Addo PG Otchere I . Antimycobacterial and cytotoxic activity of selected medicinal plant extracts. J Ethnopharmacol. (2016) 182:10–5. doi: 10.1016/j.jep.2016.02.010 26875647 PMC4801013

[18] Fraguas-Sánchez AI Torres-Suárez AI. Medical use of cannabinoids. Drugs. (2018) 78:1665–703. doi: 10.1007/s40265-018-0996-1, 30374797

[19] Riaz Z Khalid S Ali Q Hayat AN Ahmad S Haider F . Anti-microbial potential of cannabis plant extract against bacteria isolated from gut and mucus of *Oreochromis mossambicus*. Biol Clin Sci Res J. (2020):101. doi: 10.54112/bcsrj.v2022i1.101

[20] Chandra S Radwan MM Majumdar CG Church JC Freeman TP ElSohly MA. New trends in *Cannabis* potency in USA and Europe during the last decade (2008–2017). Eur Arch Psychiatry Clin Neurosci. (2019) 269:5–15. doi: 10.1007/s00406-019-00983-5, 30671616

[21] Bukowska B. Current and potential use of biologically active compounds derived from *Cannabis sativa* L. in the treatment of selected diseases. Int J Mol Sci. (2024) 25:12738. doi: 10.3390/ijms252312738 39684447 PMC11641728

[22] Eloff JN. Avoiding pitfalls in determining antimicrobial activity of plant extracts and publishing the results. BMC Complement Altern Med. (2019) 19:1–8. doi: 10.1186/s12906-019-2519-3 31113428 PMC6530048

[23] Saleemi MA Yahaya N Zain NNM Raoov M Yong YK Noor NS . Antimicrobial and cytotoxic effects of cannabinoids: an updated review with future perspectives and current challenges. Pharmaceuticals. (2022) 15:1–24. doi: 10.3390/ph15101228 PMC9607911 36297340

[24] Silva-Reis R Silva AM Oliveira PA Cardoso SM. Antitumor effects of *Cannabis sativa* bioactive compounds on colorectal carcinogenesis. Biomolecules. (2023) 13:1–21. doi: 10.3390/biom13050764 PMC10216468 37238634

[25] Zaiachuk M Pryimak N Kovalchuk O Kovalchuk I. Cannabinoids, medical *Cannabis,* and colorectal cancer immunotherapy. Front Med. (2021) 8:1–15. doi: 10.3389/fmed.2021.713153 PMC8497796 34631734

[26] Mármol I Sánchez-de-Diego C Pradilla Dieste A Cerrada E Rodriguez Yoldi MJ. Colorectal carcinoma: a general overview and future perspectives in colorectal cancer. Int J Mol Sci. (2017) 18:1–39. doi: 10.3390/ijms18010197 PMC5297828 28106826

[27] Malík M Doskočil I Pavlík J Ulman M Praus L Kouřimský P . Selective cytotoxicity of medical *Cannabis*. (*Cannabis sativa* L.) extracts across the whole vegetation cycle under various hydroponic and nutritional treatments. Cannabis and Cannabinoid Res. (2024) 9:409–20. doi: 10.1089/can.2022.0243 36459627

[28] Mthimunye LM Managa GM Nemadodzi LE. The influence of *Lablab purpureus* growth on nitrogen availability and mineral composition concentration in nutrient-poor savanna soils. Agronomy. (2023) 13:622. doi: 10.3390/agronomy13030622

[29] Eloff JN. A sensitive and quick microplate method to deter mine the minimal inhibitory concentration of plant extracts for bacteria. Planta Med. (1998) 64:711–3. doi: 10.1055/s-2006-957563, 9933989

[30] Sandasi M Leonard CM Viljoen AM. The in vitro antibiofilm activity of selected culinary herbs and medicinal plants against *Listeria monocytogenes*. Lamp. (2010) 50:30–5. doi: 10.1111/j.1472-765X.2009.02747.x, 19874481

[31] Mohsenipour Z Hassanshahian M. The effects of *Allium sativum* extracts on biofilm formation and activities of six pathogenic bacteria. J Microbiol. (2015) 8:e18971. doi: 10.5812/jjm.18971v2 PMC4600595 26464762

[32] Ahmad A Viljoen AM Chenia HY. The impact of plant volatiles on bacterial quorum sensing. Lett Appl Microbiol. (2015) 60:8–19. doi: 10.1111/lam.12343, 25346138

[33] Mosmann T. Rapid colorimetric assay for cellular growth and survival: application to proliferation and cytotoxicity assays. J Immunol Methods. (1983) 65:55–63. doi: 10.1016/0022-1759(83)90303-4 6606682

[34] Kotzé M Eloff JN. Extraction of antibacterial compounds from *Combretum microphyllum* (Combretaceae). S Afr J Bot. (2002) 68:62–7. doi: 10.1016/S0254-6299(16)30456-2

[35] Jp D Ao A Lj M Jn E. Antimycobacterial activity against different pathogens and selectivity index of fourteen medicinal plants used in Southern Africa to treat tuberculosis and respiratory ailments. S Afr J Bot. (2016) 102:70–4. doi: 10.1016/j.sajb.2015.08.002

[36] Leinen ZJ Mohan R Premadasa LS Acharya A Mohan M Byrareddy SN. Therapeutic potential of *Cannabis*: a comprehensive review of current and future applications. Biomedicine. (2023) 11:2630. doi: 10.3390/biomedicines11102630 PMC10604755 37893004

[37] Belendiuk KA Baldini LL, Bonn-Miller M.O. Narrative review of the safety and efficacy of marijuana for the treatment of commonly state-approved medical and psychiatric disorders. Addict Sci Clin Pract (2015) 10:1–10, doi: 10.1186/s13722-015-0032-7. PMC4636852 25896576

[38] Monyela S Kayoka PN Ngezimana W Nemadodzi LE. Evaluating the metabolomic profile and anti-pathogenic properties of *Cannabis* species. Meta. (2024) 14:1–26. doi: 10.3390/metabo14050253 PMC11122914 38786730

[39] Kuete V. Potential of Cameroonian plants and derived products against microbial infections: a review. Planta Med. (2010) 76:1479–91. doi: 10.1055/s-0030-1250027, 20533165

[40] Ahmed M Marrez DA Mohamed Abdelmoeen N Abdelmoneem Mahmoud E Ali MAS Decsi K . Studying the antioxidant and the antimicrobial activities of leaf successive extracts compared to the green-chemically synthesized silver nanoparticles and the crude aqueous extract from *Azadirachta indica*. PRO. (2023) 11:1644. doi: 10.3390/pr11061644

[41] Vijayalakshmi R Ambalavanan N Rajeshkumar S Mahendra J. Antimicrobial and anti-inflammatory activity of *Terminalia arjuna*. Bioinformation. (2023) 19:184–9. doi: 10.6026/97320630019184 37814683 PMC10560303

[42] Sulistiyawati TD Islamy RA. Phytochemical characteristics and antimicrobial activity of medical plant *Zizyphus mauritiana* against *Pseudomonas fluorescens*. Ecol Envir Conserv. (2021) 27:13–6.

[43] Riyadi FM Prajitno A Fadjar M Syaifurrisal A Fauziyyah AI. Potential of Moringa (*Moringa oleifera*) leaf extract to inhibit the growth of pathogenic Bacteria *Edwardsiella tarda*. JAFH. (2021) 10:321–30. doi: 10.20473/jafh.v10i3.25057

[44] Kenconojati H Ulkhaq MF Azhar MH Budi DS. Evaluation of antibacterial activity of different solvent extract from *Coffea canephora* leaves against *Edwardsiella tarda* and *Streptococcus agalactiae*. AACL Bioflux. (2019) 12:2371–7. doi: 10.22159/ajpcr.2019.v12i12.35589

[45] Marwulan M Nursyam H Kilawati Y. Antibacterial activity test of tembelekan leaf extract (*Lantana camara* Linn) against *Edwardsiella tarda* bacteria. JPPIPA. (2023) 9:3934–42. doi: 10.29303/jppipa.v9i5.3459

[46] Hakim MM Ganai NA Ahmad SM Asimi OA Raja T Shah FA . Evaluation of in vitro antioxidant activity of *Nelumbo nucifera* leaf extract and its potential application as antibacterial agent against fish pathogens. Int J Curr Microbiol Appl Sci. (2019) 8:379–89. doi: 10.20546/ijcmas.2019.806.043

[47] Syafitri E Kurniawan D Afriani DT. Antibacterial activity of guava leaf extract on the growth of *Aeromonas hydrophila*. Quagga J Pendidik Biol. (2023) 15:140–7. doi: 10.25134/quagga.v15i2.18

[48] Mutua CM Ogweno JO Gesimba RM. Effect of NPK fertilizer rates on secondary metabolites of Pepino melon (*Solanum muricatum* Aiton). J Hortic For. (2021) 13:25–34. doi: 10.5897/JHF2020.0657

[49] Famuyide IM Aro AO Fasina FO Eloff JN McGaw LJ. Antibacterial and antibiofilm activity of acetone leaf extracts of nine under investigated south African *Eugenia and Syzygium* (Myrtaceae) species and their selectivity indices. BMC Complement Altern Med. (2019) 19:1–13. doi: 10.1186/s12906-019-2547-z 31221162 PMC6587284

[50] Nguta J Kiraithe M. In vitro antimicrobial activity of aqueous extracts of *Ocimum suave* Willd*., Plectranthus barbatus* Andrews and *Zanthoxylum chalybeum* Engl. against selected pathogenic bacteria. BBRJ. (2019) 3:30–11. doi: 10.4103/bbrj.bbrj_128_18

[51] Ponnuraj S Jaganathan D Kanagarajan M Deivamarudachalam TPD. Influence of *Limonia acidissima* L. against the biofilm forming *Aeromonas hydrophila* isolated from fresh water fishes. J Biochem Technol. (2015) 6:910–21.

[52] Karthikeyan A Rameshkumar R Sivakumar N Al Amri IS Pandian SK Ramesh M. Antibiofilm activity of *Dendrophthoe falcata* against different bacterial pathogens. Biol Pharmacol Activities. (2012) 78:1918–26. doi: 10.1055/s-0032-1327879 23115018

[53] Nwanekwu KE. Evaluation of medicinal plants extract against biofilm formation in *Pseudomonas aeruginosa*. J Adv Microbiol. (2020) 20:62–6. doi: 10.9734/jamb/2020/v20i530246

[54] Alam K Al Farraj DA Mah-e-Fatima S Yameen MA Elshikh MS Alkufeidy RM . Anti-biofilm activity of plant derived extracts against infectious pathogen-*Pseudomonas aeruginosa* PAO1. J Infect Public Health. (2020) 13:1734–41. doi: 10.1016/j.jiph.2020.07.007 32753311

[55] Husain FM Ahmad I Khan MS Al-Shabib NA. *Trigonella foenum-graceum* (seed) extract interferes with quorum sensing regulated traits and biofilm formation in the strains of *Pseudomonas aeruginosa* and *Aeromonas hydrophila*. J Evid Based Complement Alternat Med. (2015) 2015:1–10. doi: 10.1155/2015/879540 PMC4427017 26000026

[56] Ibrahim HAH Abdel-Latif HH Zaghloul EH. Phytochemical composition of *Avicennia marina* leaf extract, its antioxidant, antimicrobial potentials and inhibitory properties on *Pseudomonas fluorescens* biofilm. Egypt J Aquat Res. (2022) 48:29–35. doi: 10.1016/j.ejar.2021.10.007

[57] Erhabor RC Erhabor JO Nkadimeng SM McGaw LJ. In vitro antimicrobial, antibiofilm and antioxidant activities of six South African plants with efficacy against selected foodborne pathogens. S Afr J Bot. (2022) 146:643–52. doi: 10.1016/j.sajb.2021.11.051

[58] Yüzbaşıoğlu EC Bona M Şerbetçi T Gürel F. Evaluation of quorum sensing modulation by plant extracts originating from Turkey. Int J Plant Biol. (2017) 152:376–85. doi: 10.1080/11263504.2017.1303000

[59] Taganna JC Quanico JP Perono RMG Amor EC Rivera WL. Tannin-rich fraction from *Terminalia catappa* inhibits quorum sensing (QS) in *Chromobacterium violaceum* and the QS-controlled biofilm maturation and LasA staphylolytic activity in *Pseudomonas aeruginosa*. J Ethnopharmacol. (2011) 134:865–71. doi: 10.1016/j.jep.2011.01.028, 21291979

[60] Sarabhai S Sharma P Capalash N. Ellagic acid derivatives from *Terminalia chebula* Retz. Downregulate the expression of quorum sensing genes to attenuate *Pseudomonas aeruginosa* PAO1 virulence. PLoS One. (2013) 8:e53441. doi: 10.1371/journal.pone.0053441 23320085 PMC3539995

[61] Kong J Wang Y Xia K Zang N Zhang H Liang X. New insight into the antibacterial and quorum sensing inhibition mechanism of *Artemisia argyi* leaf extracts towards *Pseudomonas aeruginosa* PAO1. Biotechnology. (2021) 11:1–15. doi: 10.1007/s13205-021-02663-5 PMC7840821 33520583

[62] Baloyi IT Adeosun IJ Yusuf AA Cosa S. Antibacterial, ant-quorum sensing, antibiofilm activities and chemical profiling of selected south African medicinal plants against multi-drug resistant bacteria. J Med Plants. (2022) 16:52–65. doi: 10.5897/JMPR2021.7192

[63] Chen Y Ma X Fu X Yan R. Phytochemical content, cellular antioxidant activity and anti-proliferative activity of *Adinandra nitida* tea (shiyacha) infusion subjected to in vitro gastrointestinal digestion. Int J Food Sci Technol. (2017) 7:50430–40. doi: 10.1039/C7RA07429H

[64] Eloff JN. On expressing the antibacterial activity of plant extracts—a small first step in applying scientific knowledge to rural primary health care. S Afr J Sci. (2000) 96:116–8.

[65] Caamal-Fuentes E Torres-Tapia L Simá-Polanco P Peraza-Sánchez SR Moo PR. Screening of plants used in Mayan traditional medicine to treat cancer-like symptoms. J Ethnopharmacol. (2011) 135:719–24. doi: 10.1016/j.jep.2011.04.004 21501677

[66] Kuete V Krusche B Youns M Voukeng I Fankam AG Tankeo S . Cytotoxicity of some Cameroonian spices and selected medicinal plant extracts. J Ethnopharmacol. (2011) 134:803–12. doi: 10.1016/j.jep.2011.01.035, 21291988

